# The efficiency of subconjunctival bevacizumab in refractory glaucoma - case report

**Published:** 2015

**Authors:** V Popescu, C Leasu, D Stana, C Alexandrescu, A Dumitrescu

**Affiliations:** * Anatomy Department, ”Carol Davila” University of Medicine and Pharmacy, Bucharest, Romania; ** Department of Clinical Ophthalmology, University Emergency Hospital Bucharest, Romania

**Keywords:** bevacizumab, glaucoma, trabeculectomy, vascular endothelial growth factor

## Abstract

Anti-vascular endothelial growth factor (anti-VEGF) therapy used as adjunctive to glaucoma filtration surgery may help filtering bleb survival because vascular endothelial growth factor has an important role in the angiogenesis of new vessels and in the fibrogenesis, which lead to scar formation and bleb failure. Bevacizumab is a non-selective monoclonal antibody against all isoforms of VEGF-A.

We present the case of an inflammatory glaucoma of a 67-year-old female, with uncontrolled intraocular pressure on maximal tolerable medical treatment, who underwent trabeculectomy and received 1.25 mg/0.05 ml of bevacizumab (Avastin) subconjunctivally at the end of the surgery and an additional injection one month later. Right eye intraocular pressure (IOP) was 26 mm Hg at preoperative visit and after surgery, it decreased and remained normal at each postoperative examination with no additional IOP-lowering medication. A localized avascular bleb with moderate elevation was observed six months postoperatively.

**Abbreviations:** VEGF = vascular endothelial growth factor, IOP = intraocular pressure, 5-FU = 5-Fluorouracil, MMC = Mitomycin-C

## Introduction

Surgical intervention in glaucoma is often done after topical treatment and laser surgery have failed to control pressure or in case of poor compliance. Trabeculectomy, the gold standard in glaucoma filtering surgery, has undergone modifications and refinements over time, with a view to improve the patient outcomes and visual recovery [**1**]. Because of the wound healing process, the success rate of filtering surgery is limited in time [**2**-**4**].

In order to reduce scar formation, antimetabolites are frequently used (5-Fluorouracil and Mitomycin-C) nowadays. The introduction of antimetabolites (5-FU and MMC) has improved the success rate and has lowered IOP compared to trabeculectomy without such agents, but the frequency of postoperative complications such as hypotony or bleb-related infections have raised [**5**-**7**].

With the aim of reducing these complications, new compounds are being investigated. Anti-vascular endothelial growth factor (anti-VEGF) therapy used as adjunctive to trabeculectomy may help filtering bleb survival because vascular endothelial growth factor has an important role in the angiogenesis of new vessels and in the fibrogenesis, which lead to scar formation and bleb failure. Bevacizumab is a non-selective monoclonal antibody against all isoforms of VEGF-A and is currently being used off-label to treat various ocular pathology, including refractory glaucoma [**8**,**9**].

## Case report

A 67-year-old woman with a history of inflammatory glaucoma presented to our department for trabeculectomy surgery without phacoemulsification due to uncontrolled IOP. This patient was under maximum tolerated medical therapy (Cosopt and Travatan). The preoperatory ophthalmologic examination included: best corrected visual acuity (BCVA) in the right eye (RE) was 20/ 60 and in the left eye (LE) was 60/ 60, intraocular pressure (IOP) using Goldmann applanation tonometry was 26 mm Hg (RE) and 19 mm Hg (LE), computerized visual field revealed superior Bjerrum scotoma in the RE, fundus examination revealed increased cup-to-disc (C/ D) ratio of 0.7 with inferior neuroretinal rim thinning and Optical Coherence Tomography found decreased RNFL in the inferior quadrant.

**Fig. 1 A-D:**
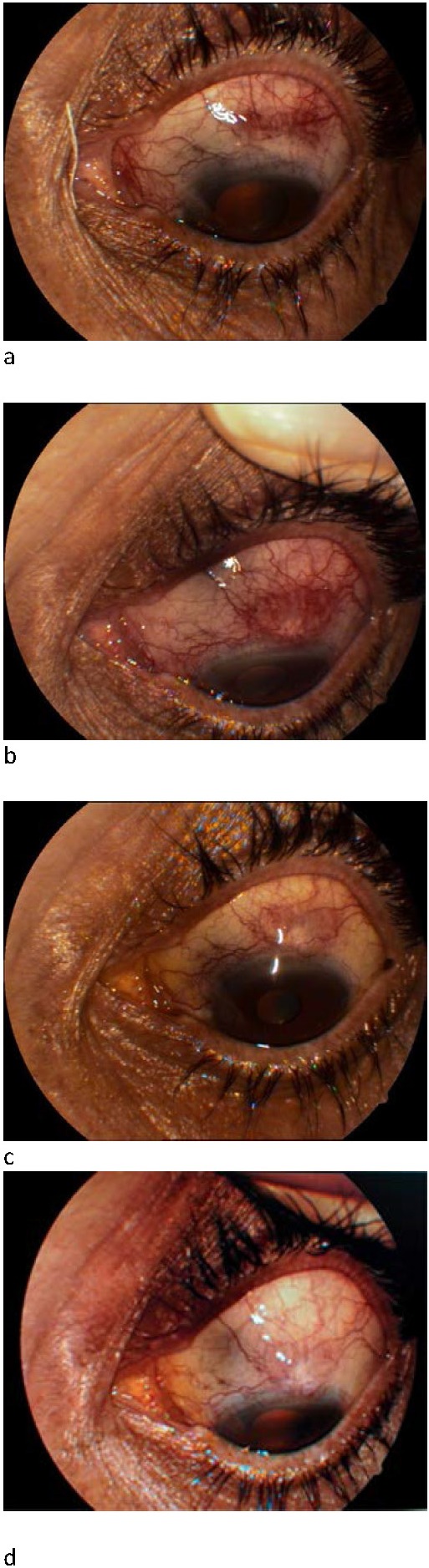
Bleb photographs recorded the evolution of bleb vascularization after subconjunctival bevacizumab injection during each follow-up visit (A: one week, B: one month, C: three months, D: six months)

A signed informed consent was obtained and trabeculectomy was performed after the administration of the peribulbar anaesthesia with lidocaine. At the end of the surgery, 1.25mg/ 0.05 ml subconjunctival bevacizumab (Avastin) was injected adjacent to the bleb by using a single-use 30-gauge needle and a tuberculin syringe. One month after the glaucoma surgery, the administration of bevacizumab injection was repeated in the temporal region of the bleb. The postoperative regimen included Netildex, Indocollyre and Tropicamida three times per day for 1 month. One day post-injection, the IOP on the RE was 15 mm Hg and at each follow-up visit did not increase more than 17 mm Hg. The final BCVA was 40/ 60. At each visit, bleb photographs were taken to evaluate the filtering bleb morphology by using the Indiana Bleb Appearance Grading Scale (**Fig. 1**). The bleb conjunctiva appeared more avascular than preoperatively, with a moderate elevation and also seemed less hyperemic than the surrounding non-operated conjunctiva.

The patient was followed-up and a full eye examination was performed one day, one week, one month, three months and six months after trabeculectomy augmented with bevacizumab.

No additional anti-glaucoma therapy was needed after surgery. No injection-related complications or drug related side effects were observed.

## Discussion

Due to the aggressive wound healing process, conventional trabeculectomy is reported to be less successful in eyes that have a pathology with poor prognosis such as inflammatory or neovascular glaucoma [**10**]. Vascular endothelial growth factor has an important role in the angiogenesis of new vessels and in the fibrogenesis, which lead to scar formation and bleb failure [**10**,**11**].

The short-term results suggested that subconjunctival injection with 1.25/ 0.05ml bevacizumab as adjunct to glaucoma filtration surgery is well tolerated and highly effective, as it was previously described [**12**-**16**].

Regarding morphological characteristics, the bleb had an observable reduction in vascularization seen on the standard photographs made with the eye looking inferiorly and interpreted by using Indiana Bleb Appearance Grading Scale (IBAGS) [**13**].

Additional follow-up and further studies on multiple patients are mandatory to evaluate the long-term efficiency of this multiple administration of bevacizumab.

## Conclusions

In this case of refractory glaucoma, bevacizumab was an useful agent for the improvement success and for the limitation of scar tissue formation after trabeculectomy.

Subconjunctival injections of bevacizumab seem to improve the function of the filtering bleb in inflammatory glaucoma because they control the bleb vascularization by blocking the angiogenesis and fibroblast modulation.

No additional anti-glaucoma therapy was needed after surgery. No injection-related complications or drug related side effects were observed.

**Acknowledgements**

This study was supported by the Sectoral Operational Programme Human Resources Development (SOPHRD), financed from the European Social Found and by the Romanian Government under the contract number POSDRU/159/1.5/S/137390 (author #1).
